# Hybrid Intrusion Detection System with Real-Time Concept Drift Detection for Enhanced IoT Security

**DOI:** 10.3390/s26165117

**Published:** 2026-08-12

**Authors:** Muath A. Obaidat, Meryem Abouali, Aneeza Shakeel

**Affiliations:** 1 John Jay College of Criminal Justice, The City University of New York, New York, NY 10019, USA; mabouali@jjay.cuny.edu; 2 Department of Cybersecurity, Tandon School of Engineering, New York University (NYU), New York, NY 10012, USA; as22329@nyu.edu

**Keywords:** IoT security, intrusion detection system, hybrid IDS, Random Forest, Isolation Forest, concept drift, Kolmogorov–Smirnov test, CICIoT2023, unseen attack evaluation

## Abstract

The rapid deployment of Internet of Things (IoT) devices across smart cities, healthcare systems, industrial automation, transportation networks, smart grids, and cyber-physical infrastructures has expanded the modern cyberattack surface. IoT devices are often constrained by limited processing capacity, memory, battery power, and communication bandwidth, making conventional security mechanisms difficult to deploy consistently at scale. Intrusion detection systems (IDSs) provide an important defensive layer; however, many machine-learning-based IDSs are developed under static assumptions and may experience performance degradation as traffic distributions evolve due to firmware changes, device onboarding, protocol updates, user behavior variation, or adaptive attacks. This paper presents a hybrid IDS framework that integrates supervised Random Forest classification, unsupervised Isolation Forest anomaly monitoring, and Kolmogorov–Smirnov (KS)-based concept drift monitoring. In the experimental pipeline, Isolation Forest is trained exclusively on benign traffic to ensure that the anomaly detector models normal behavior rather than an attack-dominated training distribution. The evaluation uses a large-scale chronologically sampled subset of the CICIoT2023 dataset containing 3,890,621 records while preserving the natural class distribution of 2.35% benign traffic and 97.65% attack traffic. The chronological 80/20 train/test split is established first at the file level, followed by systematic sampling within each split to reduce the risk of leakage across the evaluation boundary. On the 746,094-record test set, the proposed hybrid IDS achieved 99.73% accuracy, 99.89% precision, 99.83% recall, 99.86% F1-score, and a false positive rate of 4.77%. The corresponding confusion matrix contains TN = 16,683, FP = 836, FN = 1205, and TP = 727,370, yielding 95.23% specificity and 97.53% balanced accuracy. Standalone Random Forest marginally outperformed the hybrid model in raw accuracy and false positive rate; therefore, the contribution of the proposed framework is centered on deployment-oriented anomaly monitoring, drift awareness, and generalization rather than absolute superiority in static classification metrics. A leave-one-attack-family-out experiment withholding MITM-ArpSpoofing from training showed that the hybrid model detected 85.26% of the unseen attack-family samples, compared with 85.18% for Random Forest alone and 7.05% for Isolation Forest alone. These findings provide initial evidence of generalization to one held-out attack family but should not be interpreted as proof of broad zero-day detection capability. The framework is therefore positioned as a competitive IDS that combines supervised detection with anomaly monitoring and concept drift awareness for deployment-oriented IoT security.

## 1. Introduction

The Internet of Things (IoT) has become a foundational component of modern digital infrastructure. IoT devices enable continuous sensing, automation, and data exchange across healthcare, smart cities, industrial control systems, transportation, agriculture, energy distribution, smart homes, and autonomous systems. While this connectivity creates operational benefits, it also expands the cybersecurity attack surface. Unlike conventional enterprise networks, IoT environments are typically composed of heterogeneous devices with diverse hardware capabilities, operating systems, firmware versions, communication protocols, and update mechanisms. Many devices operate unattended for long periods and may be deployed in physically accessible locations, increasing exposure to compromise [1,2,3].

The constraints of IoT devices directly affect the design of security controls. Deep packet inspection, endpoint agents, complex encryption protocols, and high-overhead monitoring tools may be difficult to implement on small embedded devices. Large-scale deployments also include legacy devices, weak authentication, default credentials, limited logging, inconsistent patch management, and incomplete visibility. These weaknesses allow adversaries to conduct reconnaissance, credential attacks, malware propagation, botnet recruitment, DDoS attacks, spoofing, and command-and-control communication [1,4,5,6,7,8].

IDS technologies are commonly deployed to identify malicious behavior in network traffic. Signature-based systems are interpretable and effective against known attacks, but they are limited when attackers modify behavior or exploit new vulnerabilities. Supervised machine-learning IDSs can achieve strong performance on benchmark datasets, but they depend on representative labeled training data. Anomaly-based systems can identify deviations from normal behavior, but they may produce false positives when benign traffic changes naturally. These limitations become more serious in IoT environments because device behavior and network conditions can evolve over time [9,10,11,12,13].

A key challenge is concept drift, which occurs when the statistical properties of the data change after the model has been trained. In IoT networks, drift may arise from firmware updates, device replacement, protocol changes, new applications, network expansion, seasonal or daily usage variation, and adversarial adaptation. If an IDS is trained offline and deployed without monitoring, concept drift can increase false positives, increase false negatives, and reduce trust in the system over time [14,15,16].

Hybrid IDS research attempts to improve robustness by combining multiple detection paradigms. A supervised classifier can detect labeled attack patterns, while an unsupervised anomaly detector can monitor deviations from learned normal behavior. However, hybrid systems must be evaluated carefully. A model should not claim universal superiority if a supervised baseline achieves slightly better static accuracy. Similarly, zero-day or unknown-attack claims require a validation protocol in which selected attack families are excluded from training and used only for testing. Accordingly, this study uses the term “unseen attack-family evaluation” rather than claiming complete zero-day detection. The leave-one-attack-family-out experiment provides initial evidence of generalization to one withheld attack category, while broader zero-day detection remains a direction for future validation. To support a more rigorous evaluation, this study aligns all reported metrics with one consistent prediction output, uses a large-scale chronological evaluation, trains the Isolation Forest component on benign traffic only, and includes a leave-one-attack-family-out experiment to assess generalization to an unseen attack family [10,17,18,19,20].

The main contributions of this paper are as follows. First, it presents a modular hybrid IDS architecture that integrates Random Forest classification, benign-only Isolation Forest anomaly monitoring, and KS-based concept drift monitoring. Second, it evaluates the framework on a large-scale chronologically sampled CICIoT2023 subset that preserves the natural benign/attack imbalance instead of using an artificially balanced subset. Third, it corrects the Isolation Forest implementation by training the anomaly detector on benign traffic only, reducing the standalone benign false positive rate to 0.92%. Fourth, it provides a leave-one-attack-family-out evaluation using MITM-ArpSpoofing as a held-out family to directly test generalization to an unseen attack family. Fifth, it reframes the hybrid contribution as deployment-oriented generalization, anomaly monitoring, and drift awareness rather than raw metric superiority.

The remainder of this paper is organized as follows. Section 2 reviews related work on IoT security, IDS approaches, hybrid IDS models, and concept drift. Section 3 presents the proposed methodology. Section 4 describes the experimental setup, dataset sampling, preprocessing pipeline, model configuration, and evaluation metrics. Section 5 presents the results and discussion. Section 6 discusses limitations and future work. Section 7 concludes the paper.

## 2. Literature Review

IoT security research has evolved from device-level protections and rule-based defenses toward machine-learning and adaptive monitoring approaches that can respond to complex traffic behavior. A journal-level review should position the proposed work relative to complementary research streams rather than simply listing prior studies. This section discusses IoT threat characteristics, traditional IDS categories, hybrid IDS designs, concept drift, adaptive IDS methods, and proactive assessment techniques [1,2,3,10,14,15,16,17].

### 2.1. IoT Security Challenges and Threat Landscape

IoT networks are vulnerable at multiple layers. At the device layer, common weaknesses include default credentials, insecure firmware, absent secure boot mechanisms, inconsistent update processes, insecure storage of secrets, and limited physical protection. At the communication layer, IoT devices may use lightweight protocols such as MQTT, CoAP, Zigbee, BLE, and proprietary wireless protocols [21]. These protocols are often selected for efficiency, but insecure configuration or incomplete authentication can expose devices to spoofing, replay, routing, and man-in-the-middle attacks [22]. At the application and cloud layers, insecure APIs, weak access control, misconfigured storage, and insufficient logging can further expand the attack surface [1,2,4,5,8,23,24].

The threat landscape is also shaped by scale. In large IoT deployments, even a small vulnerability can have significant consequences because many devices may share similar firmware, libraries, credentials, or deployment patterns. Botnets such as Mirai demonstrated that poorly secured IoT devices can be compromised at scale and used to launch high-volume DDoS attacks. More recent attacks have used adaptive scanning, encrypted command-and-control traffic, and stealth-oriented reconnaissance to evade static detection rules. These trends motivate IDS designs that can detect known malicious patterns and monitor unusual behavior that deviates from expected traffic activity [6,7,25,26,27].

Large-scale IoT deployments are also vulnerable to misconfiguration, exposed services, weak authentication, and attack-surface expansion, which can be identified through proactive measurement, black-box assessment, and taxonomy-based IoT security studies [26,27,28].

### 2.2. Runtime IDS and Proactive Security Assessment

Runtime intrusion detection and proactive security assessment address different points in the IoT security lifecycle. A runtime IDS continuously monitors traffic after deployment and attempts to detect malicious or anomalous activity as it occurs. Proactive assessment, including black-box testing, vulnerability scanning, penetration testing, and pre-deployment risk analysis, identifies weaknesses before attackers exploit them. These approaches are complementary. Proactive assessment can reduce the attack surface before deployment, while a runtime IDS provides ongoing monitoring when new threats, configuration changes, or traffic shifts appear after deployment [29,30].

The proposed framework belongs primarily to runtime IDS research. It monitors traffic behavior using supervised and unsupervised learning and adds statistical monitoring for distributional changes. However, a complete IoT security strategy should combine runtime detection with proactive testing. This distinction prevents overstating the framework as a complete IoT security solution and clarifies that its role is traffic-level detection and drift monitoring after deployment [29,30].

Recent proactive IoT security studies have shown that black-box testing and device-level assessment can identify authentication, reliability, and configuration weaknesses before deployment, thereby reducing the attack surface that runtime IDS solutions must later monitor [26,27,28].

### 2.3. Signature-Based, Anomaly-Based, and Machine-Learning IDSs

Signature-based IDSs rely on predefined rules, patterns, or known indicators of compromise. They are useful in operational environments because their alerts are often interpretable and can produce low false-positive rates when signatures are accurate. However, signature-based systems are inherently limited against new malware variants, zero-day vulnerabilities, obfuscated payloads, and low-and-slow attacks that do not match existing rules. This limitation is especially important in IoT networks, where attackers can exploit device-specific weaknesses or adapt traffic behavior to evade static detection [6,7,11].

Anomaly-based IDSs model normal behavior and flag deviations as suspicious. This approach can be useful for detecting unusual activity that does not match known signatures. However, anomaly detection can be sensitive to benign variation, especially in heterogeneous IoT environments where traffic patterns differ by device type, application, time of day, and network conditions. Parameters such as anomaly threshold, contamination level, window size, and feature selection directly affect operational performance [15,16,31,32,33].

Machine-learning IDSs use labeled or unlabeled data to learn traffic patterns. Supervised models such as Random Forest, Decision Tree, Logistic Regression, and XGBoost often perform well on structured flow-based datasets because they can learn non-linear relationships among traffic features. Nevertheless, supervised models can only learn from patterns represented in training data. If future traffic differs substantially from training traffic, model performance may degrade, motivating hybrid and drift-aware IDS designs [9,10,12,13,34,35,36].

### 2.4. Hybrid Intrusion Detection Approaches

Hybrid IDS approaches combine multiple detection mechanisms to improve robustness. A common design integrates a supervised classifier with an anomaly detector. The supervised classifier provides high-confidence detection for attack patterns represented in labeled data, while the anomaly detector monitors deviations from expected behavior. Other hybrid systems combine deep learning feature extraction with classical classifiers, ensemble methods with thresholding, or signature rules with statistical anomaly detection [10,17,18,19,20,37].

The main advantage of hybrid design is broader detection coverage, but it also introduces fusion-rule trade-offs. A simple OR rule can increase recall but may increase false positives. An AND rule can reduce false positives but may miss attacks. A confidence-based conditional rule can balance these goals by allowing the anomaly detector to influence the final decision when supervised confidence is uncertain. In this study, the hybrid model is used as a deployment-oriented monitoring framework rather than as a claim of absolute metric superiority, because standalone Random Forest marginally outperforms the hybrid model in raw accuracy and false positive rate on the current evaluation.

### 2.5. Concept Drift and Adaptive IDS

Concept drift is a key challenge in streaming and dynamic environments. In IDSs, drift means that the feature distribution or the relationship between features and labels changes over time. If a model is trained offline and deployed without monitoring, drift can cause future traffic to be misclassified. In IoT environments, drift may occur naturally through device, firmware, protocol, and user-behavior changes, or adversarially through attacker adaptation [14,15,16,38].

Existing drift-aware IDS approaches include periodic retraining, online learning, incremental updates, ensemble adaptation, sliding-window monitoring, feature-rank monitoring, statistical hypothesis testing, and adaptive stream-learning frameworks [15,16,38,39,40,41,42,43,44,45,46,47]. Periodic retraining is simple but may retrain unnecessarily or miss sudden shifts. Online and incremental learning can adapt continuously, but these methods must be controlled to avoid learning from poisoned or malicious data [45]. Feature-drift and adaptive sliding-window methods provide additional ways to monitor evolving streams, while statistical tests provide lightweight and interpretable signals that indicate when a distribution has changed. The KS test is useful because it is non-parametric and compares empirical cumulative distribution functions without assuming a specific underlying distribution [14,48].

In this paper, the KS test is used as a monitoring mechanism rather than as proof of automatic adaptation. The KS module identifies distributional shifts between a reference traffic window and a current traffic window. When drift is detected, the system can trigger human-supervised model review or controlled retraining. Automatic retraining is not claimed unless the full retraining pipeline is implemented and experimentally validated [14,44,48].

In Table 1, the comparison clarifies the position of the proposed work. The framework is not presented as a replacement for all IDS methods. Instead, it integrates complementary capabilities in one pipeline: supervised Random Forest detection for known patterns, benign-only Isolation Forest anomaly monitoring for deviations from normal traffic, and KS-based monitoring for distributional changes. This combination is particularly relevant for IoT environments where traffic evolves and where highly complex deep learning models may not always be practical for edge-assisted deployment.

### 2.6. Research Gap

The reviewed literature suggests four gaps. First, many IDS studies emphasize static classification performance while providing limited discussion of deployment-oriented factors such as false positives, drift monitoring, class imbalance, reproducibility, and explainability [10,51,52]. Second, hybrid IDS studies do not always define fusion rules clearly or analyze their effect on recall and false positives [17,18]. Third, concept drift is often discussed conceptually without a lightweight monitoring mechanism integrated into the IDS pipeline [14,15,16]. Fourth, claims of zero-day detection are sometimes made without an unseen-attack validation protocol. This study addresses these gaps by using a consistent large-scale chronological evaluation, correcting the anomaly-monitoring component, adding explicit KS-based drift monitoring, and testing generalization with a leave-one-attack-family-out experiment.

## 3. Proposed Methodology

This section presents the proposed hybrid IDS framework. The system is designed as a modular pipeline that separates preprocessing, supervised classification, anomaly monitoring, drift monitoring, decision fusion, and retraining signaling. This modular design allows each component to be evaluated independently while supporting integration into a broader IoT security monitoring architecture.

### 3.1. System Architecture

The proposed system consists of five stages: data collection and flow representation, preprocessing, hybrid detection, concept drift monitoring, and retraining-trigger generation. Raw IoT traffic is converted into structured flow-level features. The preprocessed feature vectors are evaluated by the Random Forest classifier and the Isolation Forest anomaly monitor. Their outputs are combined through a defined fusion rule to produce the final benign-versus-attack decision. In parallel, the KS drift module monitors feature distributions across time windows and generates a controlled model-review signal if significant distributional change is detected [10,14,17,33,48].

**Figure 1:**
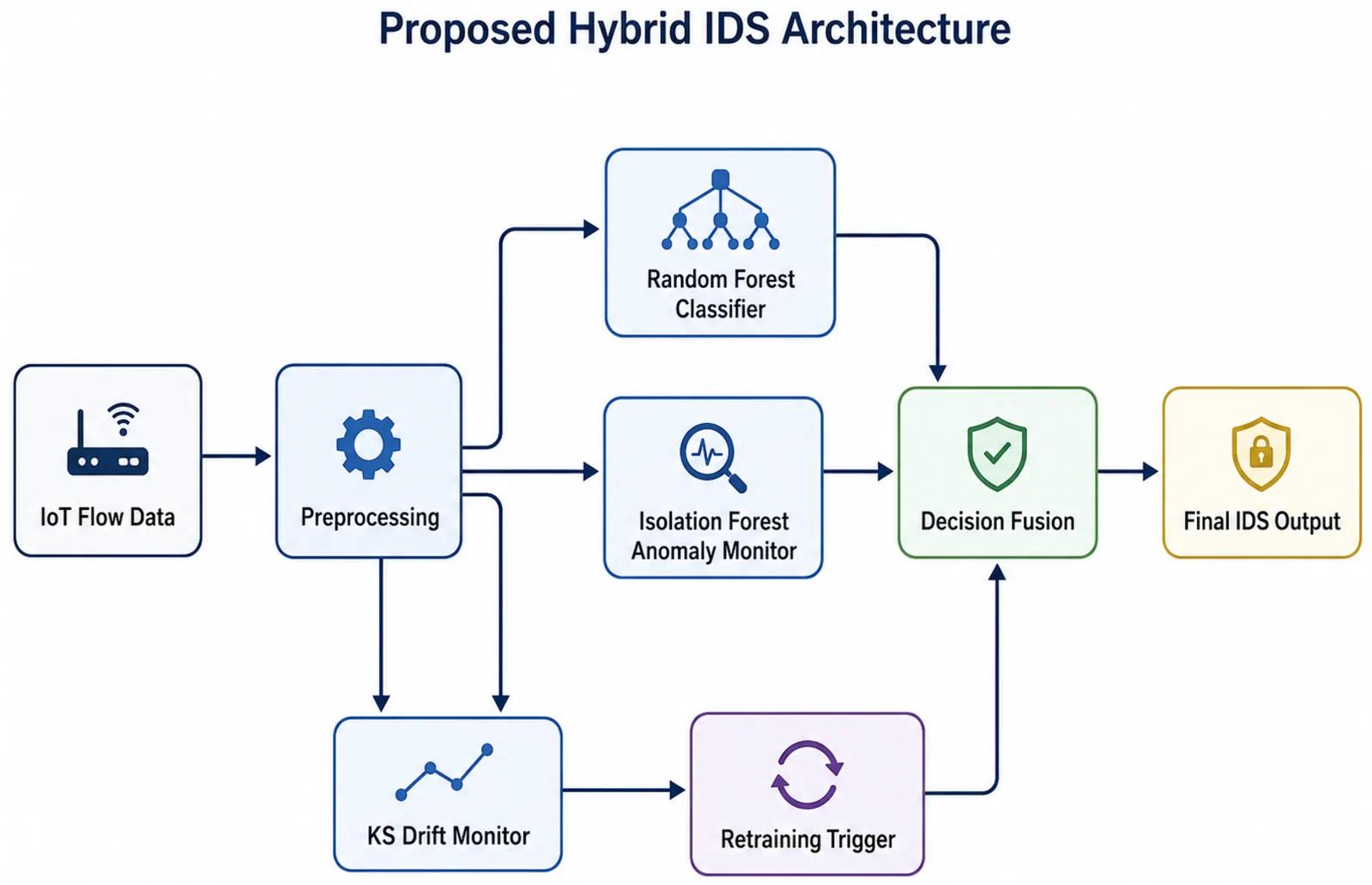
Proposed hybrid IDS architecture integrating Random Forest classification, benign-only Isolation Forest anomaly monitoring, decision fusion, and KS-based drift monitoring.

The architecture intentionally separates drift detection from automatic retraining. The KS component functions as a drift-monitoring and retraining-trigger mechanism. A safe online retraining system would require reliable sample selection, protection against poisoning, model update validation, and deployment safeguards. Therefore, automatic retraining is treated as future work rather than claimed as a completed capability. As illustrated in Figure 1.

### 3.2. Data Preprocessing

Preprocessing ensures that the supervised, unsupervised, and statistical monitoring components operate on consistent input representations. The experimental pipeline removes non-informative identifiers, handles missing or invalid values through zero imputation, converts multiclass labels into binary labels, and applies Z-score standardization fitted on the training data only. Benign traffic is labeled as 0, and malicious traffic is labeled as 1, making Attack = 1 the positive class [53,54,55].

All 46 original numerical features are retained, and no feature-selection step is applied. Z-score standardization is fitted on the training set only and then applied to the test set to avoid leakage from test statistics. This distinction is important because fitting scalers or preprocessing transformations on the full dataset can leak information from the evaluation set into training. The same preprocessing pipeline is used for all evaluated models so that performance differences reflect model behavior rather than inconsistent data preparation [53,54,56,57,58].

### 3.3. Random Forest Classification

The supervised component uses Random Forest classification to identify known attack patterns represented in labeled training data. Random Forest constructs an ensemble of decision trees using bootstrap sampling and randomized feature selection. Each tree learns a set of decision rules, and the final prediction is obtained through aggregation across trees. This ensemble design reduces variance compared with a single decision tree and improves robustness to noisy features [34,35,36,53,59].

Random Forest is well suited to structured IoT flow data because it can model non-linear relationships among packet counts, byte statistics, timing features, protocol attributes, and flag-related variables. It is also computationally practical compared with many deep neural architectures and provides feature-importance estimates that support interpretability. In the proposed framework, Random Forest provides supervised evidence that a traffic instance resembles known benign or malicious behavior learned from the training set [34,35,53,59].

### 3.4. Isolation Forest Anomaly Monitoring

The unsupervised component uses Isolation Forest to monitor anomalous behavior. Isolation Forest is based on the principle that anomalies are easier to isolate than normal observations because they are relatively rare and different. The model builds random partitioning trees and assigns anomaly scores based on the average path length required to isolate a sample. Shorter path lengths indicate greater abnormality [31,32,33].

Training Isolation Forest on the full training set is not appropriate in this evaluation because the training data is dominated by attack traffic, with approximately 97.6% of records representing attacks. Under this setting, the anomaly detector may learn attack behavior as normal behavior, which reduces its validity as a normal-behavior anomaly monitor. Therefore, the implemented pipeline trains Isolation Forest only on benign training samples, which is consistent with standard anomaly-detection design. This benign-only training strategy reduced the standalone benign false positive rate to 0.92%, substantially improving interpretability and operational validity.

The contamination parameter controls the expected proportion of anomalies and therefore affects the Isolation Forest decision threshold. A high contamination value can cause the model to flag too many benign samples as anomalous, increasing false positives, while a low contamination value may reduce sensitivity to subtle malicious behavior. The contamination sweep evaluates values of 0.005, 0.01, and 0.02, and the final configuration uses 0.01 as a reasonable middle ground between accuracy and false positive rate [31,32,33,60,61].

### 3.5. Hybrid Decision Fusion

The decision fusion rule defines how Random Forest and Isolation Forest outputs are combined. In a hybrid IDS, fusion can be designed to prioritize recall, false-positive reduction, or balanced performance. A union-based rule labels a traffic instance as malicious if either the supervised classifier predicts attack or the anomaly detector flags it as anomalous. This rule can reduce false negatives but may increase false positives if the anomaly detector is overly sensitive [10,17,18].

In the revised results, the hybrid model is not presented as universally superior to standalone supervised models. Instead, the hybrid contribution is framed around complementary monitoring and generalization. Random Forest provides strong labeled-pattern detection, while benign-only Isolation Forest adds a label-free anomaly signal. The leave-one-attack-family-out experiment shows that the hybrid model slightly improves detection of an unseen attack family compared with Random Forest alone, although the improvement is modest. This framing is more scientifically defensible than claiming absolute superiority in raw metrics.

### 3.6. KS-Based Concept Drift Monitoring

The concept drift component uses the two-sample Kolmogorov–Smirnov test to compare the empirical distribution of feature values in a reference window with the distribution in a current monitoring window. For a feature x, the KS statistic is defined as

(1) $$D=\underset{x}{\sup}\left|F_{\mathrm{ref}}\left(x\right)-F_{\mathrm{cur}}\left(x\right)\right|,$$

where $F_{\mathrm{ref}}\left(x\right)$ and $F_{\mathrm{cur}}\left(x\right)$ are the empirical cumulative distribution functions of the reference and current traffic windows, respectively. The null hypothesis states that both samples are drawn from the same distribution. If the *p*-value is below the significance threshold α, the null hypothesis is rejected, and the feature is considered to show evidence of distributional shift [14,48].

Because IoT datasets contain multiple features, declaring drift based on a single feature may be unstable. The framework treats drift as a multi-feature monitoring event and analyzes all 46 retained numerical features. To maximize chronological separation, the earliest training samples are compared with the latest test samples. No statistically significant drift is detected across the 46 features, with all *p*-values greater than 0.19. This result is interpreted as a property of CICIoT2023, which was captured in a short controlled testbed window, not as a failure of the KS mechanism. The KS statistics are non-degenerate and vary across features, confirming that the implementation is functioning correctly [14,15,16,48].

## 4. Experimental Setup

This section describes the dataset, sampling design, preprocessing pipeline, model configuration, evaluation metrics, and reproducibility details. The experiment uses a large-scale chronologically sampled subset of CICIoT2023 containing 3,890,621 records while preserving the natural 2.35% benign class distribution. This design supports evaluation under realistic class imbalance and provides detailed information needed for reproducibility. This design strengthens the manuscript by aligning the evaluation with realistic class imbalance.

### 4.1. Dataset Description

The CICIoT2023 dataset is used as the source dataset for evaluating the proposed framework [62,63]. The full dataset contains 46,686,579 records distributed across 169 chronologically ordered files. The dataset includes benign IoT traffic and multiple attack families, including DDoS, DoS, reconnaissance, web-based attacks, brute-force attempts, spoofing, and Mirai-related attacks [63]. In this study, the original labels are converted to a binary classification problem in which benign traffic is labeled 0 and all malicious traffic is labeled 1 [23,64,65,66,67,68,69,70].

### 4.2. Large-Scale Chronological Sampling Design

The evaluation uses systematic sampling rather than random or balanced sampling. Specifically, every 12th row is retained within the 169 chronologically ordered CICIoT2023 source files. The chronological 80/20 train/test split is determined first at the file level, with 139 files assigned to training and the remaining 30 files assigned to testing based on cumulative row count. Systematic sampling is then applied separately within the training and testing files. This order of operations prevents leakage across the train/test boundary because no sampled test records influence training selection.

The final subset contains 3,890,621 records, including 3,144,527 training records and 746,094 testing records. The subset preserves the dataset’s natural class imbalance: 91,595 benign records (2.35%) and 3,799,026 attack records (97.65%). This is more realistic than an artificially balanced subset and allows the manuscript to discuss false-positive behavior under a class distribution closer to the original dataset, as illustrated in Table 2.

### 4.3. Preprocessing Pipeline

The preprocessing pipeline includes missing-value handling, label binarization, and feature standardization. Missing values are handled through zero imputation. All attack families are mapped to the positive class, Attack = 1, while benign traffic is mapped to Benign = 0. All 46 original numerical features are retained, and no feature-selection step is applied. Z-score standardization is fitted only on the training data and then applied to the test data to avoid leakage [12,53,54,55].

### 4.4. Model Configuration

The evaluated models include Random Forest, XGBoost, Decision Tree, Logistic Regression, standalone benign-only Isolation Forest, and the proposed hybrid RF + IF framework. The Random Forest, XGBoost, Decision Tree, and Logistic Regression models serve as supervised baselines. Isolation Forest serves as the anomaly-monitoring component and is trained only on benign training samples. The hybrid model combines supervised classification with anomaly monitoring and integrates KS-based drift monitoring as an additional deployment-oriented capability [35,36,53,71,72,73,74], as shown in Table 3.

### 4.5. Experimental Environment and Reproducibility

The experimental environment is reported to support reproducibility. The dataset is the CICIoT2023 full dataset obtained through its original distribution and a Kaggle mirror of the UNB CIC IoT 2023 dataset [62,63]. The implementation was developed in Python 3.12 using scikit-learn 1.5.1, XGBoost 3.3.0, NumPy 1.26.4, and Pandas 2.2.2. All models’ initialization processes use a random seed of 42. The row sampling is deterministic because it uses systematic every-12th-row sampling. Experiments were run on the PSC Bridges-2 GPU-shared partition with 5 CPU cores, approximately 22.75 GB of allocated RAM, and an NVIDIA V100-32 GPU allocated but not used because all models are CPU-based. The experimental code and processed subset-generation scripts are available from the corresponding author upon reasonable request, as shown in Table 4.

### 4.6. Experimental Workflow

The experimental workflow follows a structured pipeline designed to approximate realistic IoT deployment conditions while maintaining reproducibility and avoiding data leakage [23,64,66,69]. The process begins with the CICIoT2023 source files, which are first organized according to their chronological order. An 80/20 train/test split is then performed at the file level, with 139 files assigned to the training partition and 30 files assigned to the testing partition. After the split boundary is fixed, systematic sampling is applied independently within the training and testing partitions by retaining every 12th row. This procedure produces a large-scale chronologically sampled subset while preserving the natural class imbalance of the dataset.

After sampling, the raw network-flow records undergo preprocessing, including missing-value handling, binary label conversion, and Z-score feature standardization. Benign traffic is labeled as 0, while all attack categories are labeled as 1. Missing values are handled using zero imputation, and all 46 original numerical features are retained. Z-score standardization is fitted only on the training data and then applied to the test data to prevent information leakage. The resulting feature vectors are then used to train and evaluate the supervised and unsupervised components of the proposed hybrid IDS framework [53,54,55,65].

The Random Forest classifier is trained on the labeled training data to learn known benign and malicious traffic patterns. In contrast, the Isolation Forest anomaly detector is trained only on benign training samples. This correction is important because training the Isolation Forest on the full training set, which is dominated by attack traffic, can cause the model to learn attack behavior as normal. Training the Isolation Forest on benign-only samples provides a more appropriate anomaly-detection baseline and improves the interpretability of anomaly scores [31,32,33].

During testing, each traffic instance is evaluated by both the Random Forest classifier and the Isolation Forest anomaly detector. The Random Forest component provides a supervised prediction based on learned decision boundaries, while the Isolation Forest component provides anomaly-oriented evidence based on deviation from benign behavior. The outputs of both components are combined using the hybrid decision-fusion logic to produce the final IDS classification output [10,17,18].

In parallel with the classification process, the KS-based concept drift monitoring module compares feature distributions across chronological windows. Specifically, the two-sample Kolmogorov–Smirnov test is used to compare empirical feature distributions between reference and current traffic windows. This module is used to identify statistically significant distributional shifts that may indicate changing traffic behavior. In the current implementation, the KS module provides a drift-monitoring and retraining-trigger signal; it does not perform fully automatic retraining. Any model update is treated as a controlled or human-supervised process rather than an autonomous adaptation mechanism [14,15,16,48].

**Figure 2:**
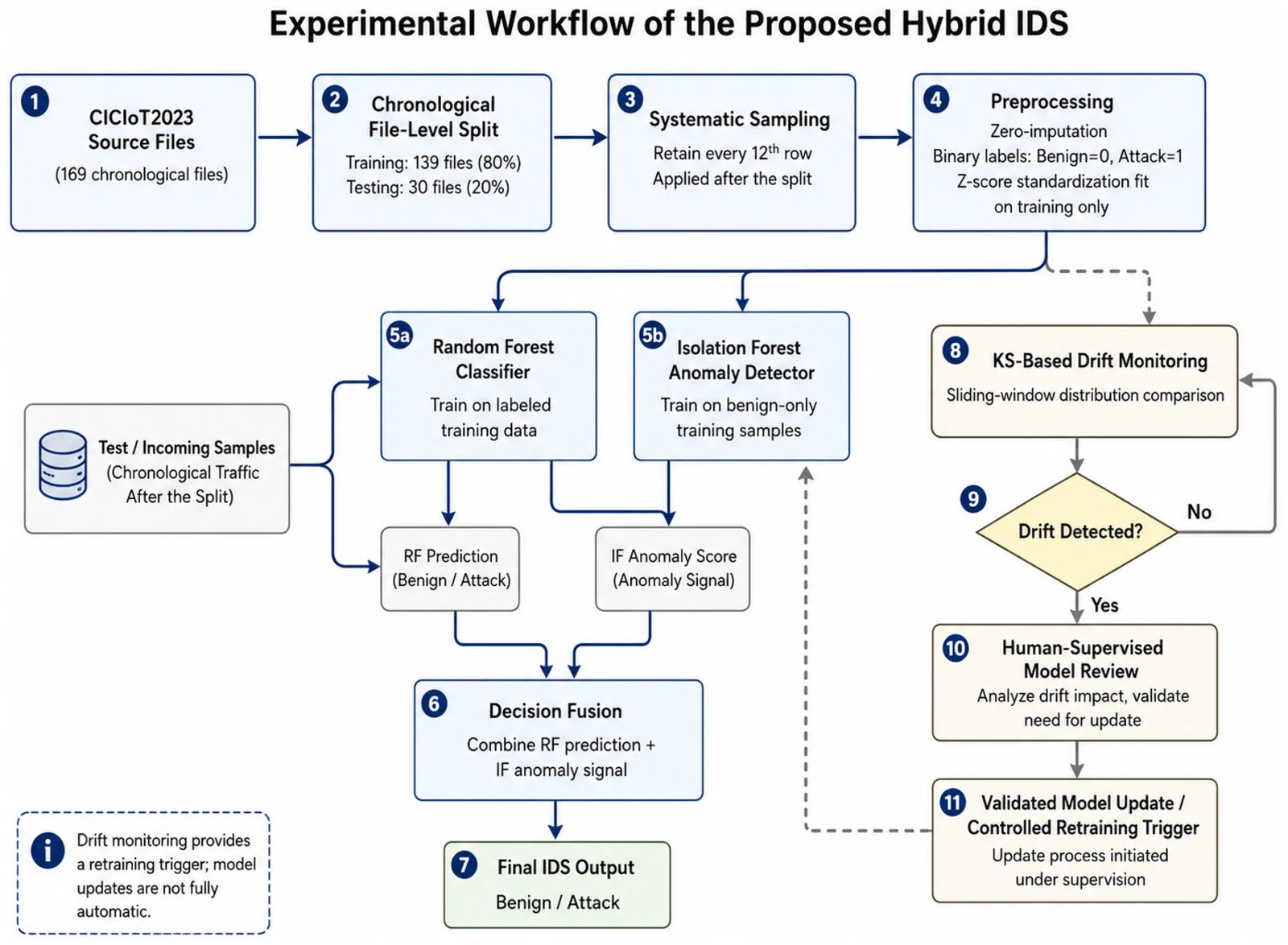
Experimental workflow of the proposed hybrid IDS framework, including chronological splitting, systematic sampling, preprocessing, Random Forest classification, benign-only Isolation Forest anomaly monitoring, decision fusion, and KS-based drift monitoring.

The overall workflow is illustrated in Figure 2. This workflow ensures that data splitting, sampling, preprocessing, detection, fusion, and drift monitoring are performed in a consistent and reproducible sequence.

### 4.7. Evaluation Metrics

The positive class is defined as Attack = 1, and the negative class is defined as Benign = 0. True positives are attack records correctly classified as attacks. True negatives are benign records correctly classified as benign. False positives are benign records incorrectly classified as attacks. False negatives are attack records incorrectly classified as benign. Because the dataset is highly imbalanced, accuracy alone is not sufficient. The evaluation therefore reports precision, recall, F1-score, false positive rate, specificity, and balanced accuracy.. The performance of the proposed intrusion detection framework is assessed using the evaluation metrics summarized in Table 5.

## 5. Results and Discussion

This section reports the revised large-scale chronological-subset results. All model metrics, tables, and figures are computed from one consistent saved set of predictions. This resolves the earlier inconsistency between the confusion matrix, precision, false positive rate, and performance tables. The discussion also reframes the hybrid model appropriately: a standalone Random Forest marginally achieves the strongest raw accuracy and false positive rate, while the hybrid model provides competitive performance and additional evidence of modest generalization support in the unseen attack-family experiment.

### 5.1. Hybrid Confusion Matrix Analysis

**Figure 3:**
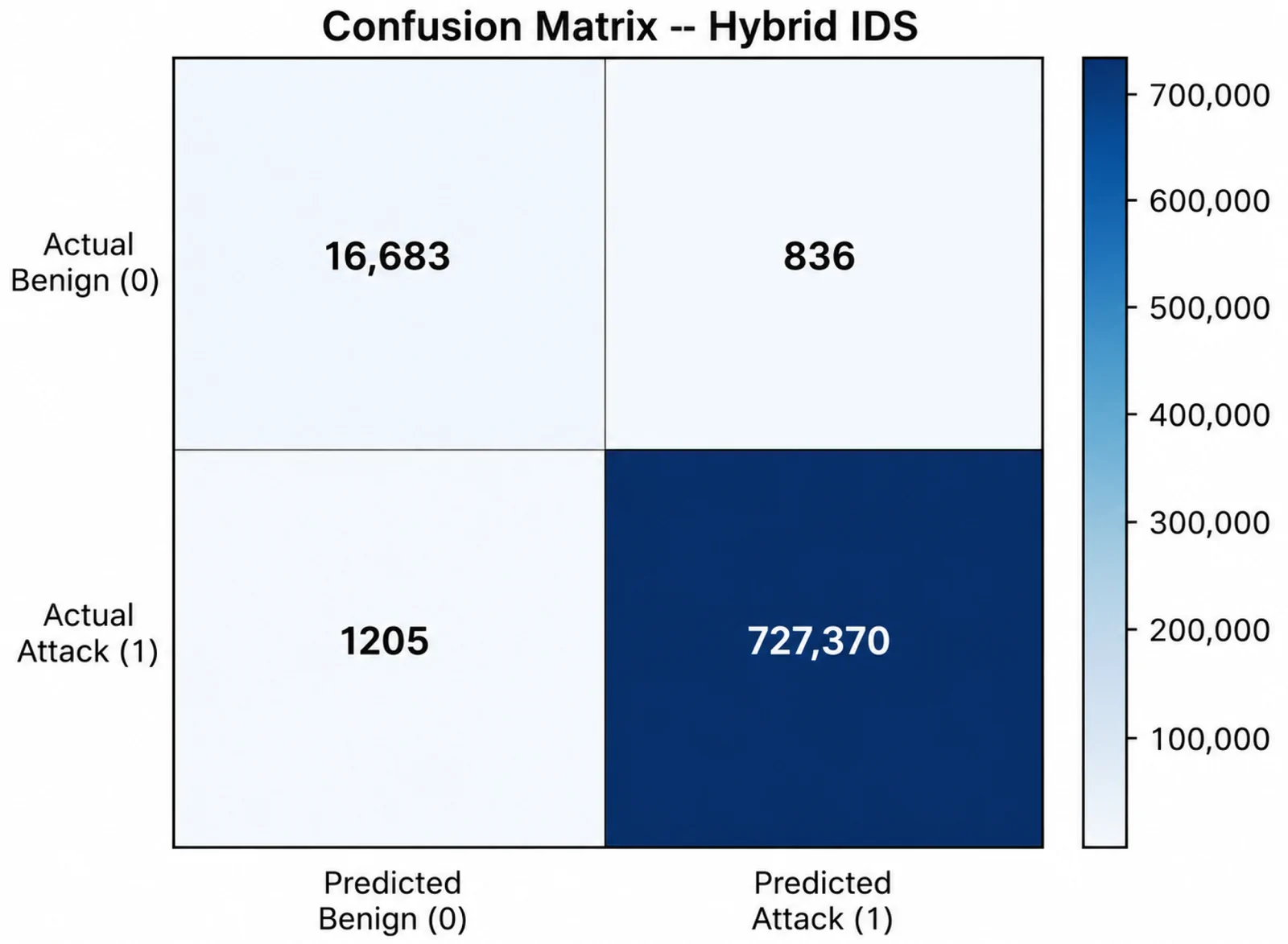
Confusion A matrix of the proposed hybrid IDS on the large chronologically sampled CICIoT2023 test set.

Figure 3 presents the confusion matrix for the hybrid IDS on the 746,094-record test set. The model correctly classifies 16,683 benign records and 727,370 attack records. It misclassifies 836 benign records as attacks and 1205 attack records as benign. These values produce 99.73% accuracy, 99.89% precision, 99.83% recall, 99.86% F1-score, and a 4.77% false positive rate. Specificity is 95.23%, and balanced accuracy is 97.53%.

The false positive rate is operationally meaningful because it represents benign traffic that would generate alerts. Although the test set is dominated by attack traffic, the benign class contains 17,519 records, allowing false-positive behavior to be measured directly. The hybrid model flags 836 benign records as attacks, corresponding to a false positive rate of 4.77%. This rate is higher than Random Forest alone but substantially lower than Logistic Regression. The results demonstrate strong attack detection while showing that the hybrid fusion rule introduces a modest false-positive trade-off.

### 5.2. Performance Comparison of Models

**Figure 4:**
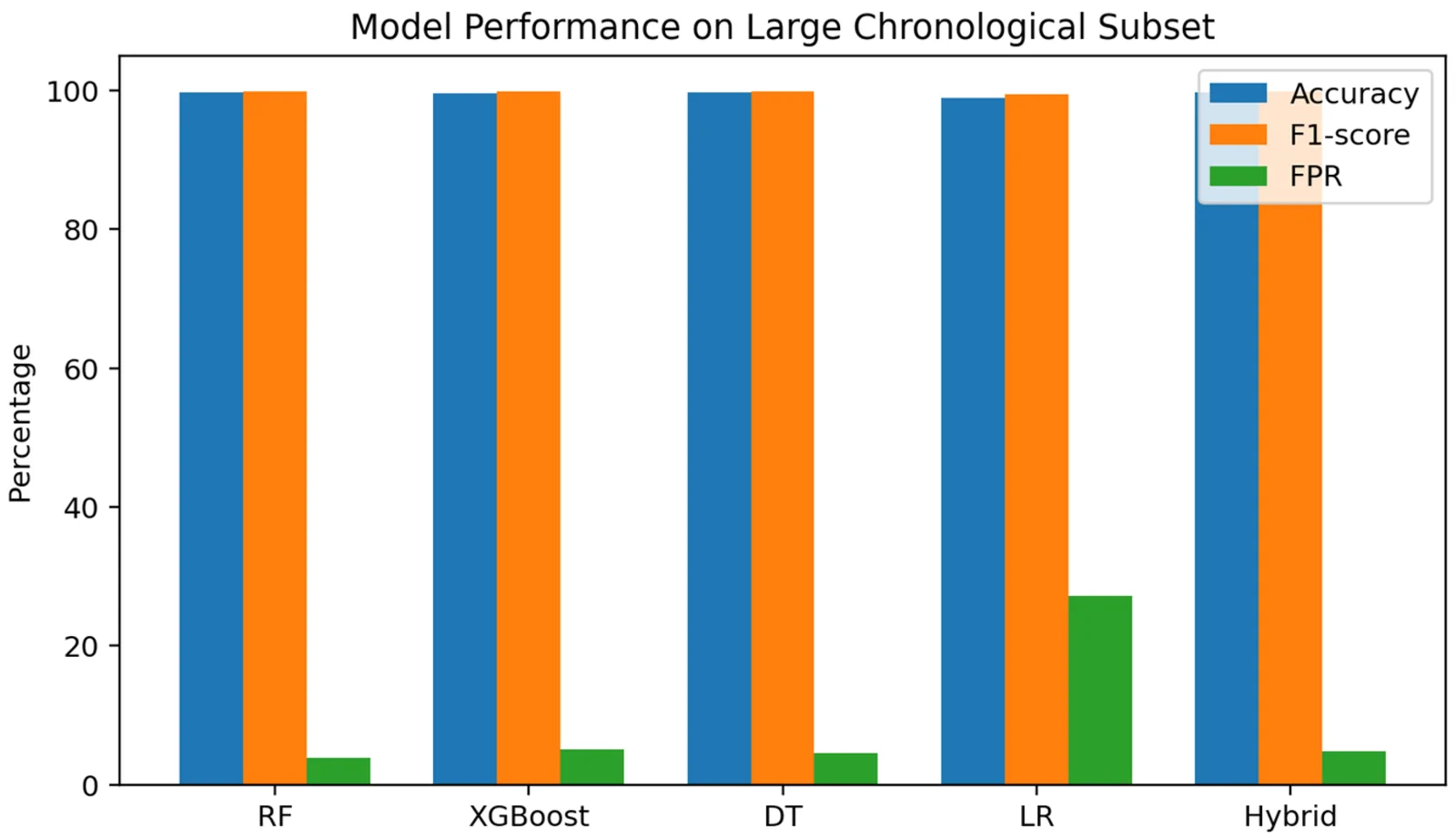
Model performance comparison on the large chronological subset.

Table 6 compares the hybrid IDS with the supervised baselines using the same preprocessing procedure and a large chronological test split. Random Forest achieves the highest accuracy (99.75%) and F1-score (99.87%), as well as the lowest false positive rate among the reported supervised and hybrid models (3.87%). The hybrid IDS achieves 99.73% accuracy and a 99.86% F1-score, which are only slightly below the corresponding Random Forest results. These findings demonstrate that the hybrid IDS remains highly competitive, although it does not clearly outperform Random Forest in terms of raw static classification metrics. Standalone Isolation Forest is evaluated as a benign-only anomaly monitor rather than as a fully supervised binary classifier. Under this design, its benign false positive rate is 0.92%, indicating that the anomaly detector can model normal traffic behavior without excessively flagging benign samples. This result is discussed in Section 5.3 as part of the corrected benign-only Isolation Forest training strategy. Table 6 provides the numerical comparison of the evaluated models, while Figure 4 visually summarizes their performance on the large chronological subset.

The results show that supervised tree-based models perform strongly on known attack patterns in CICIoT2023. Therefore, the proposed hybrid IDS is not presented as outperforming all baseline models in raw classification metrics. Instead, it is positioned as a deployment-oriented framework that integrates supervised detection, benign-only anomaly monitoring, and KS-based drift monitoring. This framing is consistent with the experimental evidence and avoids overstating the contribution of the hybrid model.

### 5.3. Corrected Isolation Forest Training Strategy

Training Isolation Forest on the full training set is not appropriate in this evaluation because the training data is dominated by attack traffic. Under this setting, the anomaly detector may incorrectly treat attack behavior as normal behavior. Therefore, Isolation Forest is trained exclusively on benign training samples. This design is consistent with standard anomaly detection, where the model learns the structure of normal traffic and flags deviations as anomalous [31,32,33].

After applying benign-only training, standalone Isolation Forest achieves a benign false positive rate of 0.92%. This improves the methodological validity of the hybrid system because the anomaly component now represents deviations from benign behavior rather than deviations from an attack-dominated mixture.

### 5.4. Contamination Parameter Sensitivity

**Figure 5:**
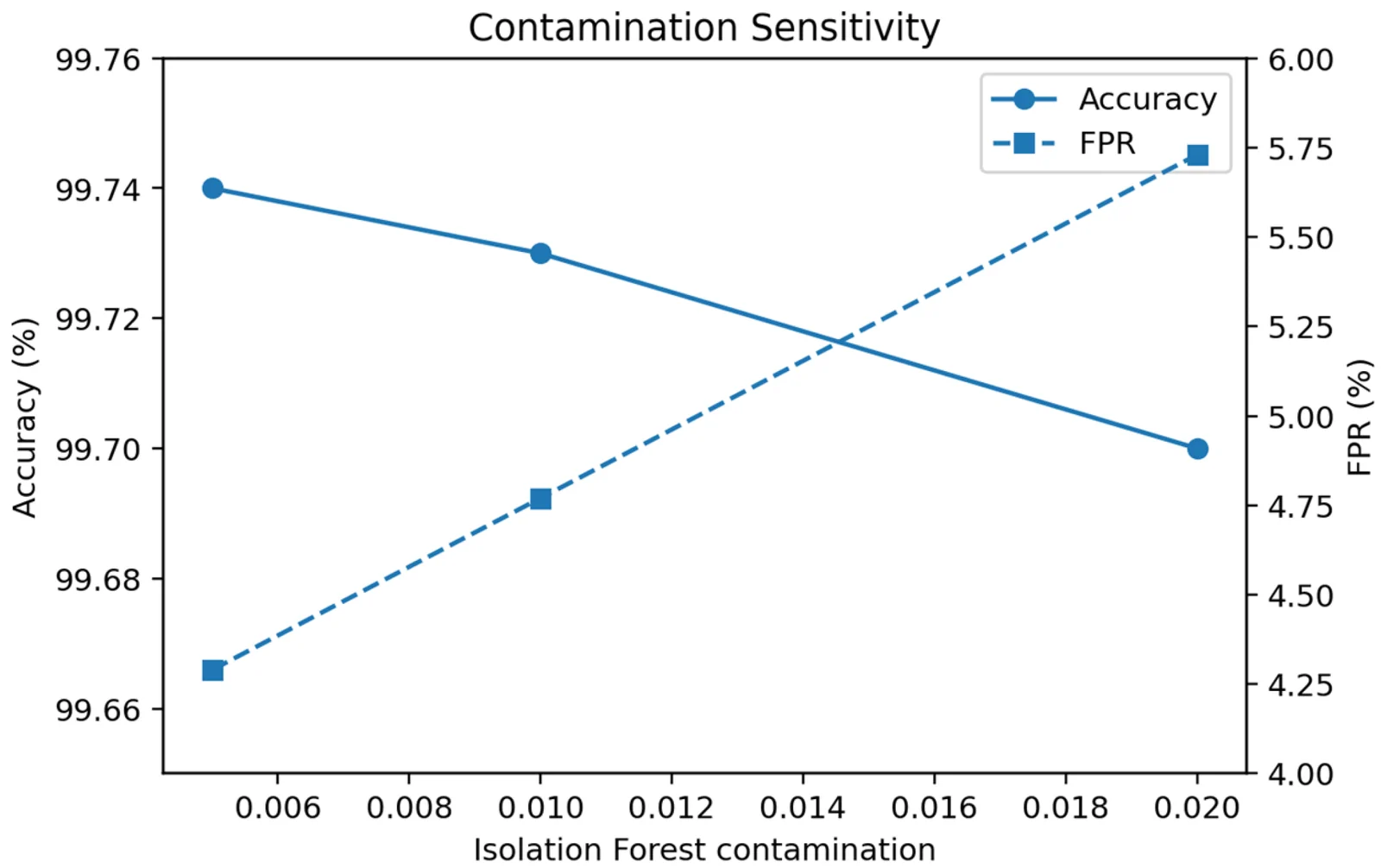
Contamination sensitivity showing the trade-off between accuracy and false positive rate.

The contamination parameter controls the expected proportion of anomalies in Isolation Forest. The contamination sweep evaluates the effect of different Isolation Forest contamination values on accuracy and false positive rate. The corrected sweep reports results for contamination levels of 0.005, 0.01, and 0.02, showing the expected trend: lower contamination yields a lower false-positive rate, while higher contamination increases the rate of anomaly flags. The final setting uses contamination = 0.01 as a middle ground between accuracy and false positive rate. The sensitivity of the hybrid IDS to the Isolation Forest contamination parameter is summarized in Table 7 and illustrated in Figure 5. As the contamination level increases from 0.005 to 0.020, accuracy decreases slightly from 99.74% to 99.70%, while the false positive rate increases from 4.29% to 5.73%. These results indicate that a lower contamination setting provides the most favorable balance between overall accuracy and false positive behavior in the evaluated hybrid pipeline.

This result has practical implications. In security operations, a lower false positive rate may be preferred if analyst workload is the main constraint. In high-risk environments, a slightly higher contamination value may be acceptable if the goal is to maximize anomaly sensitivity. The selected value should therefore be justified by deployment requirements rather than chosen only to maximize accuracy.

### 5.5. Concept Drift Monitoring Results

The KS-based drift analysis compares the earliest training samples against the latest test samples to maximize chronological separation. Across all 46 features, no statistically significant drift is detected, with 0/46 features showing significance and all *p*-values greater than 0.19. This result is consistent with CICIoT2023 being captured in a short, controlled testbed window rather than over an extended real-world deployment period.

Importantly, the absence of statistically significant drift should not be interpreted as a failure of the KS mechanism. The implementation was verified to produce varying, non-degenerate KS statistics across features, which indicates that the previous flat-plot issue was not caused by a broken statistical test. Instead, the dataset itself appears to provide limited real drift within the sampled chronological window. For future work, the drift module should be tested on data collected over longer real-world deployment periods or on synthetic drift scenarios where the time and magnitude of distributional shift are known.

### 5.6. Unseen Attack-Family Evaluation

**Figure 6:**
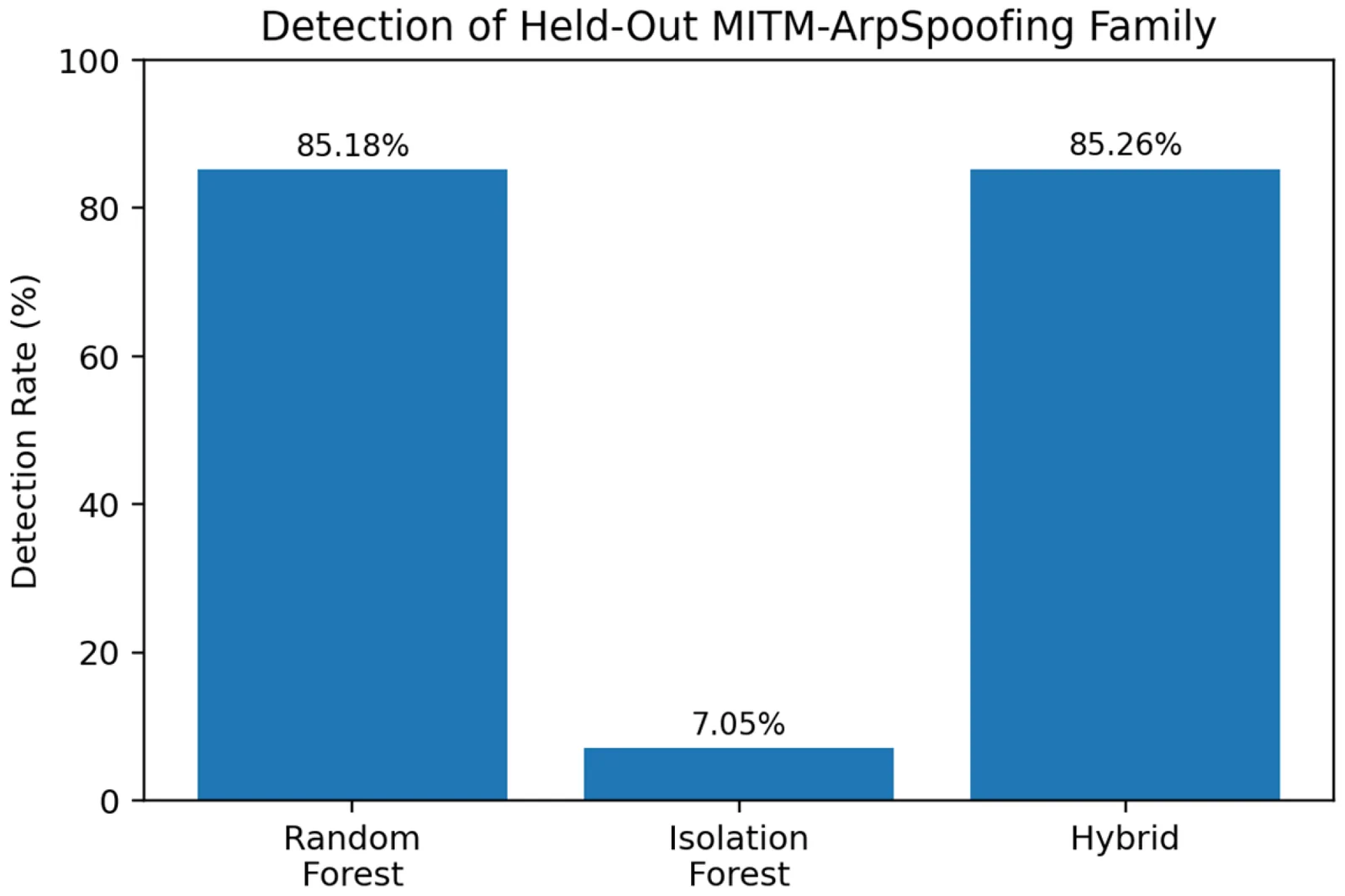
Detection rate on the held-out MITM-ArpSpoofing attack family.

To evaluate generalization beyond attack patterns observed during supervised training, the study includes a leave-one-attack-family-out experiment. The MITM-ArpSpoofing family is fully excluded from training, removing 20,601 training records associated with this family. The trained models are then evaluated specifically on 4952 test instances from the withheld MITM-ArpSpoofing family. This design tests whether the models can detect an attack family that was not included during supervised training. The leave-one-attack-family-out evaluation on MITM-ArpSpoofing is summarized in Table 8 and illustrated in Figure 6. Random Forest alone achieves a detection rate of 85.18%, whereas Isolation Forest alone detects only 7.05% of the held-out attack family. The hybrid RF + IF configuration achieves the highest detection rate of 85.26%, representing only a marginal improvement over Random Forest alone. These results indicate that the supervised Random Forest component remains the primary contributor to unseen-family detection in this experiment.

These results should be interpreted as initial evidence of generalization to a held-out attack family rather than as a complete validation of zero-day detection. Because only one attack family was withheld, additional leave-one-family-out experiments involving structurally different attack categories, such as web-based attacks, reconnaissance, brute-force attacks, and DDoS variants, are needed to validate broader unknown-attack detection.

The known-attack test performance remains essentially stable when MITM-ArpSpoofing is excluded from training. Overall accuracy on remaining known attacks is 99.67%, compared with 99.73% in the baseline run, and the false positive rate is 3.66%, compared with 4.77% in the baseline run. These results suggest that the leave-one-family-out exclusion does not substantially damage the general known-attack performance, while providing a more defensible test of generalization than the original binary-only evaluation.

### 5.7. Practical Deployment Implications

The revised large-scale results provide a more realistic view of deployment behavior because the sampled subset preserves the natural class imbalance of CICIoT2023. In a highly imbalanced IDS setting, high precision may coexist with a meaningful false-positive rate because the attack class dominates the test set. For deployment, the false positive rate and specificity are especially important because they indicate how many benign events will generate alerts.

The proposed framework is most appropriate for deployment at an IoT gateway, edge server, or network-monitoring node rather than on every constrained endpoint. The gateway can aggregate flow-level features, apply Random Forest and Isolation Forest models, and maintain reference windows for KS-based monitoring. This architecture reduces the computational burden on individual IoT devices while providing centralized security visibility. Future deployment studies should measure runtime overhead, memory consumption, model update cost, and detection latency [13,21,22].

## 6. Limitations and Future Work

Several limitations remain. First, although the experiment uses a large-scale 3,890,621-record subset, it does not evaluate all 46,686,579 records in the full CICIoT2023 dataset. The systematic sampling strategy preserves chronological order and the natural class distribution; however, rare attack subtypes may still be underrepresented. Future work should evaluate the complete dataset or compare results across multiple independently sampled subsets.

Second, the leave-one-attack-family-out evaluation tested only one held-out attack family: MITM-ArpSpoofing. Although this design is stronger than the original binary-only evaluation, it does not fully prove broad zero-day detection. The results should therefore be interpreted as initial evidence of unseen attack-family generalization. Future work should repeat this evaluation across additional and structurally distinct attack families, including web-based attacks, reconnaissance, brute-force attacks, and DDoS variants.

Third, the KS drift module was verified as functioning correctly, but CICIoT2023 did not show statistically significant drift across the tested chronological window. Future work should evaluate the drift module on longer-term real deployment data or controlled synthetic-drift scenarios with known drift points. This would allow quantitative measurement of drift-detection latency, sensitivity, and false drift alarm rate.

Fourth, the system detects drift but does not automatically perform retraining in the current implementation. Retraining is intended as a human-supervised or scheduled process. A safe automated retraining system must address data trust, poisoning risk, validation before deployment, rollback mechanisms, and post-update monitoring. Future work should implement and evaluate such a pipeline before claiming fully autonomous adaptation.

Fifth, the current evaluation uses binary classification, so the system identifies traffic as benign or malicious but does not identify the specific attack type. In practice, different attack families require different response actions. Future work should extend the framework to multiclass or hierarchical detection, where binary alerting is followed by attack-family classification [23,64,66,67,68,69].

## 7. Conclusions

This paper presented a hybrid intrusion detection framework that integrates Random Forest classification, benign-only Isolation Forest anomaly monitoring, and KS-based concept drift detection for IoT environments. Unlike many IDS studies that emphasize static accuracy, this work focuses on deployment-oriented capabilities: supervised detection of known attacks, label-free anomaly monitoring, and interpretable drift signaling.

The framework was evaluated on a large-scale chronologically sampled subset of CICIoT2023 (3.89 million records), preserving the natural 2.35% benign class imbalance. The hybrid model achieved competitive performance (99.73% accuracy, 99.86% F1, 4.77% FPR), while the standalone Random Forest marginally outperformed it in raw metrics. Rather than claiming universal superiority, the hybrid value lies in its modular integration of complementary detection signals.

The leave-one-attack-family-out experiment showed modest generalization to unseen MITM-ArpSpoofing traffic (85.26% detection), suggesting the framework can detect structurally similar attack behavior beyond its training labels. However, this should not be interpreted as proven zero-day capability—broader validation across multiple withheld families remains necessary.

The KS drift module was verified as functional, though CICIoT2023 showed no significant drift across its controlled collection window. Future work should apply the drift monitor to longer-term real deployments or synthetic scenarios with known shift points. Additional priorities include evaluating the full 46-million-record dataset, extending to multiclass classification, and developing a safely automated retraining pipeline.

Overall, this work shows that a hybrid IDS with integrated drift awareness can provide strong, generalizable detection while maintaining operational transparency—a practical step toward more resilient IoT security monitoring.

## Figures and Tables

**Table 1:** Comparative summary of IDS approaches and the proposed hybrid framework.

IDS Approach	Detection Strategy	Main Strength	Main Limitation	Drift/IoT Suitability
Signature-based IDS [6,7,11]	Matches known attack rules/signatures.	Interpretable and accurate for known attacks.	Weak against new or evolving attacks.	Lightweight but static; no native drift handling.
Supervised ML IDS [9,10,12,13]	Classifies traffic using labeled data.	Strong performance on represented attack patterns.	Depends on labels and stable distributions.	Suitable for gateways; requires retraining under drift.
Unsupervised anomaly IDS [31,32,33]	Learns normal behavior and flags deviations.	Useful when attack labels are limited.	Can create false positives from benign variation.	Efficient models are suitable, but thresholds need tuning.
Deep hybrid IDS [19,20]	Combines deep feature learning and classification.	Captures complex spatial/temporal patterns.	Higher computational cost.	Better for edge/cloud than constrained endpoints.
Drift-aware IDS [14,48,49]	Monitors feature or distribution shifts.	Detects evolving traffic behavior.	Often not a complete classifier.	Useful when integrated with IDS decision logic.
Periodic retraining IDS [1,15,16,50]	Updates the model at fixed intervals.	Reduces long-term degradation.	May retrain too late or unnecessarily.	Practical but less adaptive than drift-triggered review.
Proposed hybrid IDS	Integrates RF, benign-only IF, and KS monitoring.	Combines known-attack detection, anomaly monitoring, and drift awareness.	RF slightly outperforms hybrid in raw metrics; retraining is not automatic.	Suitable for IoT gateway/edge-assisted monitoring with controlled retraining triggers.

**Table 2:** Large-scale chronologically sampled subset composition.

Class Label	Class Meaning	Total Samples	Training Samples	Test Samples	Percentage
0	Benign traffic	91,595	74,076	17,519	2.35%
1	Attack traffic	3,799,026	3,070,451	728,575	97.65%
Total	Chronological subset	3,890,621	3,144,527	746,094	100.00%

**Table 3:** Model configuration summary.

Model	Role in Evaluation	Key Configuration/Note
Random Forest	Supervised baseline and hybrid component	Strong tree-based classifier for structured flow data; random_state = 42.
XGBoost	Supervised baseline	Gradient-boosted tree model for structured features; random_state = 42.
Decision Tree	Supervised baseline	Single-tree reference model for comparison with ensemble methods.
Logistic Regression	Linear baseline	Provides a simpler linear reference point.
Isolation Forest	Standalone anomaly detector and hybrid component	Trained on benign-only samples; contamination = 0.01 in the main hybrid run.
Hybrid RF + IF	Proposed hybrid IDS	Combines Random Forest prediction with benign-only Isolation Forest anomaly monitoring.

**Table 4:** Experimental environment and reproducibility details.

Component	Description
Dataset	CICIoT2023 full dataset: 46,686,579 records across 169 files, via Kaggle mirror of the UNB CIC IoT 2023 dataset.
Evaluation setting	Large-scale chronologically sampled subset preserving natural class distribution; not balanced.
Total samples	3,890,621 total records: 3,144,527 training and 746,094 testing.
Benign samples	91,595 total: 74,076 training and 17,519 testing; 2.35% of total.
Attack samples	3,799,026 total: 3,070,451 training and 728,575 testing; 97.65% of total.
Sampling method	Systematic sampling; every 12th row retained within 169 chronologically ordered source files.
Train/test split	80/20 chronological split at file level: 139 files for training and 30 files for testing. Sampling was applied after the split.
Label encoding	Benign = 0; Attack = 1.
Number of features	46 original numerical features retained; no feature selection applied.
Preprocessing	Missing values handled with zero imputation; labels binarized; all 46 original numerical features retained; Z-score standardization fitted on the training set only, then applied to the test set to avoid leakage.
Programming language and libraries	Python 3.12; scikit-learn 1.5.1; XGBoost 3.3.0; NumPy 1.26.4; Pandas 2.2.2.
Random seed	42 for model initialization; sampling is systematic/deterministic.
Hardware	PSC Bridges-2, GPU-shared partition; 5 CPU cores; approximately 22.75 GB RAM; NVIDIA V100-32 GPU allocated but not used because models are CPU-based.
Code availability	Available upon request.

**Table 5:** Evaluation metrics used to assess the performance of the proposed intrusion detection framework.

Metric	Formula	Interpretation
Accuracy	(TP+TN)/(TP+TN+FP+FN)	Overall proportion of correct classifications.
Precision	TP/(TP+FP)	Among predicted attacks, the proportion that are true attacks.
Recall	TP/(TP+FN)	Among actual attacks, the proportion detected.
F1-score	2×(Precision×Recall)/(Precision+Recall)	Harmonic mean of precision and recall.
False Positive Rate	FP/(FP+TN)	Proportion of benign samples incorrectly flagged as attacks.
Specificity	TN/(TN+FP)	Proportion of benign samples correctly classified.
Balanced Accuracy	(Recall+Specificity)/2	Mean of attack-detection and benign-detection performance.

**Table 6:** Performance comparison of models on the large chronologically sampled CICIoT2023 test set.

Model	Accuracy	Precision	Recall	F1-Score	FPR
Random Forest	99.75%	99.91%	99.83%	99.87%	3.87%
XGBoost	99.62%	99.88%	99.73%	99.81%	5.01%
Decision Tree	99.69%	99.89%	99.79%	99.84%	4.56%
Logistic Regression	98.89%	99.35%	99.51%	99.43%	27.20%
Hybrid (RF + IF)	99.73%	99.89%	99.83%	99.86%	4.77%

**Table 7:** Isolation Forest contamination sweep in the hybrid pipeline.

Contamination	Accuracy	FPR
0.005	99.74%	4.29%
0.010	99.73%	4.77%
0.020	99.70%	5.73%

**Table 8:** Leave-one-attack-family-out evaluation on MITM-ArpSpoofing.

Component	Detection Rate on Held-Out MITM-ArpSpoofing Family
Random Forest alone	85.18%
Isolation Forest alone	7.05%
Hybrid (RF + IF)	85.26%

## Data Availability

The CICIoT2023 dataset used in this study is publicly available through its original distribution and Kaggle mirror. The experimental code and scripts used for preprocessing, systematic subset generation, model training, and evaluation can be made available from the corresponding author upon reasonable request.
